# The Paraventricular Thalamic Nucleus and Its Projections in Regulating Reward and Context Associations

**DOI:** 10.1523/ENEURO.0524-23.2024

**Published:** 2024-02-09

**Authors:** Dillon S. McDevitt, Quinn W. Wade, Greer E. McKendrick, Jacob Nelsen, Mariya Starostina, Nam Tran, Julie A. Blendy, Nicholas M. Graziane

**Affiliations:** ^1^Neuroscience Program, Penn State College of Medicine, Hershey, Pennsylvania 17033; ^2^Department of Anesthesiology and Perioperative Medicine, Penn State College of Medicine, Hershey, Pennsylvania 17033; ^3^Doctor of Medicine Program, Penn State College of Medicine, Hershey, Pennsylvania 17033; ^4^Department of Systems Pharmacology and Translational Therapeutics, Perelman School of Medicine, University of Pennsylvania, Philadelphia, Pennsylvania 19104; ^5^Departments of Anesthesiology and Perioperative Medicine and Pharmacology, Penn State College of Medicine, Hershey, Pennsylvania 17033

**Keywords:** conditioned place preference, motivation, nucleus accumbens shell, opioids, paraventricular thalamic nucleus, reward

## Abstract

The paraventricular thalamic nucleus (PVT) is a brain region that mediates aversive and reward-related behaviors as shown in animals exposed to fear conditioning, natural rewards, or drugs of abuse. However, it is unknown whether manipulations of the PVT, in the absence of external factors or stimuli (e.g., fear, natural rewards, or drugs of abuse), are sufficient to drive reward-related behaviors. Additionally, it is unknown whether drugs of abuse administered directly into the PVT are sufficient to drive reward-related behaviors. Here, using behavioral as well as pathway and cell-type specific approaches, we manipulate PVT activity as well as the PVT-to-nucleus accumbens shell (NAcSh) neurocircuit to explore reward phenotypes. First, we show that bath perfusion of morphine (10 µM) caused hyperpolarization of the resting membrane potential, increased rheobase, and decreased intrinsic membrane excitability in PVT neurons that project to the NAcSh. Additionally, we found that direct injections of morphine (50 ng) in the PVT of mice were sufficient to generate conditioned place preference (CPP) for the morphine-paired chamber. Mimicking the inhibitory effect of morphine, we employed a chemogenetic approach to inhibit PVT neurons that projected to the NAcSh and found that pairing the inhibition of these PVT neurons with a specific context evoked the acquisition of CPP. Lastly, using brain slice electrophysiology, we found that bath-perfused morphine (10 µM) significantly reduced PVT excitatory synaptic transmission on both dopamine D1 and D2 receptor–expressing medium spiny neurons in the NAcSh, but that inhibiting PVT afferents in the NAcSh was not sufficient to evoke CPP.

## Significance Statement

This study investigates the direct impact of paraventricular thalamic nucleus (PVT) inhibition on reward-related behaviors, employing manipulations related to drugs of abuse, specifically morphine, as well as employing chemogenetic approaches that replicate the inhibitory effects induced by morphine. Our findings reveal that morphine exerts an inhibitory effect on PVT neurons projecting to the nucleus accumbens shell (NAcSh). Furthermore, local administration of morphine within the PVT elicits reward-related behaviors, a response mimicked by the inhibition of PVT neurons projecting to the NAcSh. These results firmly establish the PVT as an integral component of a complex neurocircuit involved in the acquisition of associations with opioid-related contexts. Additionally, these results provide compelling evidence linking the inhibition of PVT neurons to reward processes.

## Introduction

The paraventricular thalamic nucleus (PVT) regulates motivation, reward, aversion, and arousal via excitatory projections that extend throughout the reward network (Kelley et al., 2005; Browning et al., 2014; Matzeu et al., 2014; Kirouac, 2015; Millan et al., 2017; Zhou and Zhu, 2019; McGinty and Otis, 2020; Curtis et al., 2021; De Groote and de Kerchove d'Exaerde, 2021; Hartmann and Pleil, 2021; Iglesias and Flagel, 2021; Penzo and Gao, 2021). One particularly dense projection from the PVT is to the nucleus accumbens shell (NAcSh; Li and Kirouac, 2008). These fibers innervate both dopamine D1 and D2 receptor–expressing medium spiny neurons (D1-MSNs and D2-MSNs, respectively; Zhu et al., 2016; Li et al., 2018) with evidence suggesting that these inputs, when activated, signal stress, aversion, and arousal (Zhu et al., 2016; Ren et al., 2018; Otis et al., 2019). For example, PVT neurons that project to the NAcSh display increased c-*fos* expression levels following acute stress (Bubser and Deutch, 1999). Additionally, footshock or restraint stress exposure reduces inhibitory synaptic transmission onto PVT neurons up to 24 h poststress (Beas et al., 2018). Furthermore, activating PVT excitatory presynaptic terminals in the NAcSh evokes behavioral aversion and induces conditioned place aversion (Zhu et al., 2016; Giannotti et al., 2021), whereas heroin administration, which is strongly rewarding, reliably reduces PVT-to-NAc activity (Vollmer et al., 2022).

Negative affective states play a key role in triggering the desire to consume and seek drugs of abuse (Koob, 2008, 2020; Koob and Moal, 2008). It has been shown that optogenetic activation of the PVT-to-NAc pathway, which drives aversive states (Zhu et al., 2016; Giannotti et al., 2021), significantly increases active lever presses for heroin in a cue-induced reinstatement session (Giannotti et al., 2021). In contrast, inhibition of PVT-to-NAc projections, which would theoretically decrease aversive states, prevents heroin reinstatement (Giannotti et al., 2021). Consistent with this, Keyes et al. (2020) used a conditioned place preference (CPP) paradigm to test how inhibiting the PVT-to-NAc pathway affects morphine-induced CPP. Using CPP, Keyes et al. conditioned mice with systemic injections of morphine (context 1) or saline (context 2). Following these conditioning sessions, place preference for the morphine-paired context was measured during drug abstinence. It was shown that mice expressed a strong place preference for the morphine-paired context. However, inhibition of PVT-to-NAc excitatory synaptic transmission blocked morphine-induced CPP (Keyes et al., 2020).

Overall, these results provide evidence that the PVT-to-NAc pathway, when activated, drives aversive states while, when inhibited, decreases aversive states. However, it remains unknown whether opioids directly act within PVT neurocircuits to drive reward-related phenotypes. Additionally, it remains unknown whether direct inhibition of the PVT-to-NAc pathway in the absence of other stimuli is sufficient to promote reward.

The PVT expresses elevated levels of µ-opioid receptors (MORs; Mansour et al., 1995; Ding et al., 1996; Kolaj et al., 2014; Mengaziol et al., 2022). Application of DAMGO, the selective MOR agonist, induces hyperpolarization of PVT neurons, decreases PVT neuron input resistance, and activates an inwardly rectifying potassium conductance in the presence of tetrodotoxin (Brunton and Charpak, 1998; Goedecke et al., 2019). Additionally, using brain slice electrophysiology, bath application of DAMGO suppresses excitatory transmission from the PVT to downstream targets such as the amygdala (Goedecke et al., 2019). Based on these findings, the PVT-to-NAcSh neurocircuit may be a critical pathway that regulates opioid-related reward behaviors. Here, we examine MOR-related effects on the PVT-to-NAcSh neurocircuit using behavioral as well as pathway and cell-type specific approaches.

## Materials and Methods

### Animals

**Figure 1.**
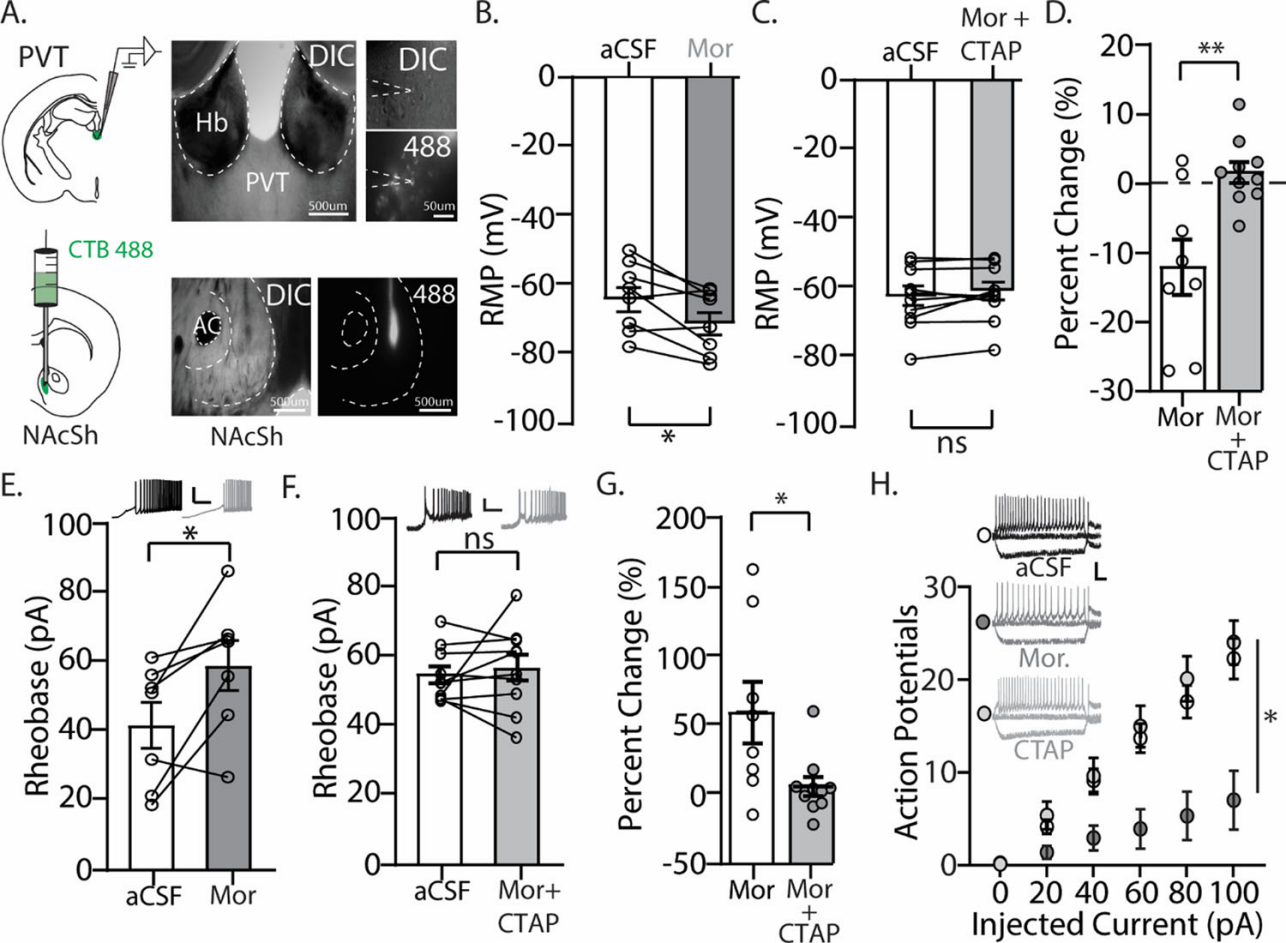
Bath application of morphine hyperpolarizes PVT neurons that project to the NAcSh in C57BL/6 wild-type mice. ***A***, (Left) Illustration of CTB injections in the NAcSh with recordings taking place in the PVT. (Right) DIC and fluorescent images showing CTB injections in the NAcSh and fluorescently labeled neurons in the PVT. ***B***, Graph showing the RMP of individual PVT neurons that project to the NAcSh before (aCSF) and after (Mor) bath application of morphine (10 µm; 8 neurons/5 mice: 3 males, 2 females). ***C***, Graph showing the RMP of individual PVT neurons that project to the NAcSh before (aCSF) and after (Mor + CTAP) bath application of morphine (10 µm) in the presence of CTAP (1 µm; 10 neurons/3 male mice). ***D***, Graph showing the percent decrease in the RMP from baseline following morphine or Mor + CTAP. ***E***, Representative traces (top) and graph showing the rheobase of individual neurons before and after bath application of morphine (10 µm; 9 neurons/5 mice: 3 males, 2 females). Scale bars: 40 mV, 800 ms. ***F***, Representative traces (top) and graph showing the rheobase of individual neurons before and after bath application of Mor (10 µm) + CTAP (1 µm; 10 neurons/3 male mice). Scale bars: 25 mV, 250 ms. ***G***, Graph showing the percent decrease in the rheobase from baseline following morphine or Mor + CTAP. ***H***, Representative traces (left) and summary graph showing the number of action potentials fired in PVT neurons that project to the NAcSh is significantly decreased following bath application of morphine (10 µm), which was blocked in the presence of CTAP. Scale bars: 25 mV, 100 ms. aCSF (open circles): *n* = 11 neurons/5 mice (3 males, 2 females). Mor (dark gray circles): *n* = 7 neurons/5 mice (3 males, 2 females). Mor + CTAP (light gray circles): *n* = 9 neurons/3 male mice. *p < 0.05; **p < 0.01.

**Figure 2.**
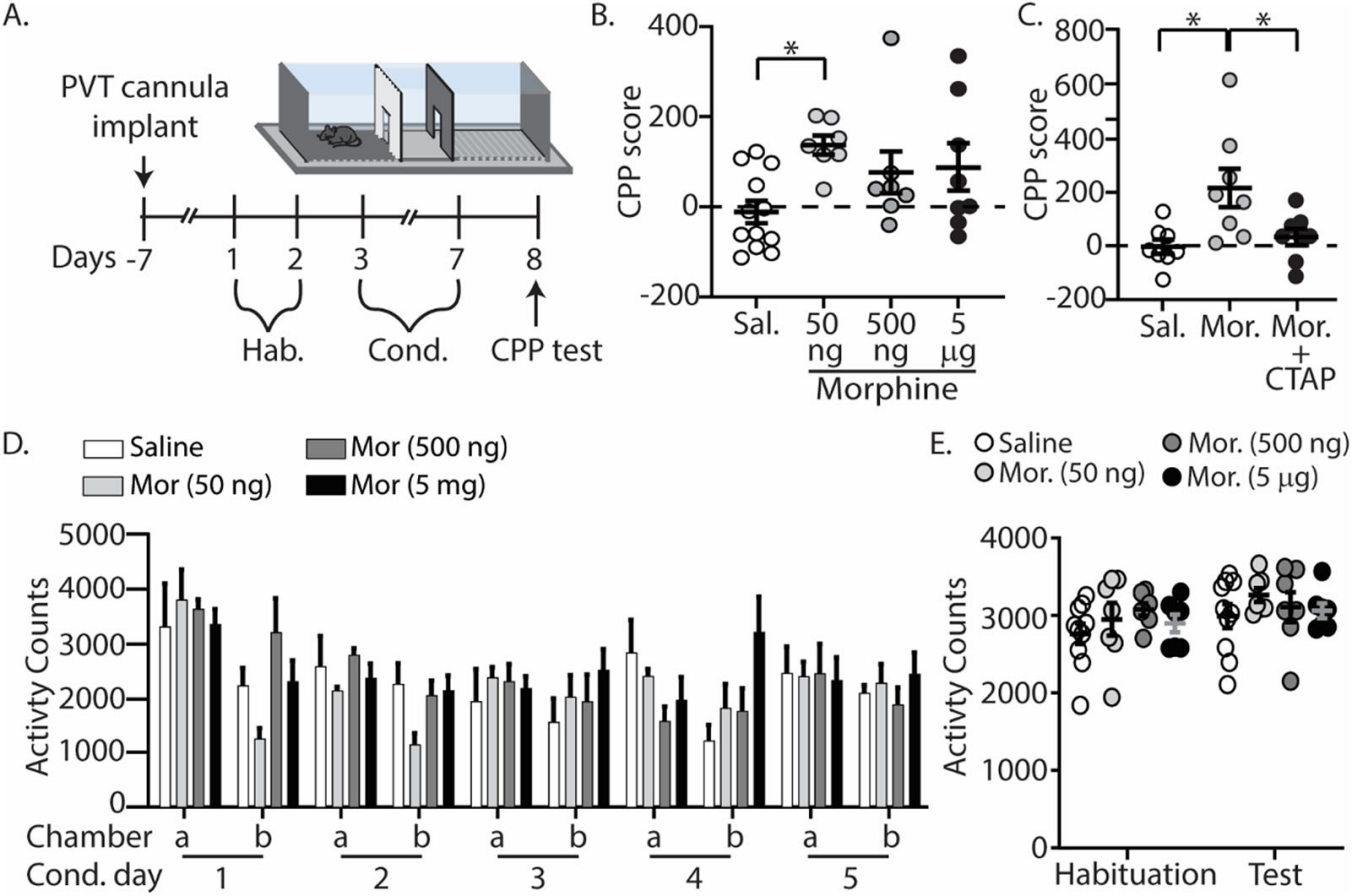
Direct application of morphine in the PVT permits CPP. ***A***, Experimental timeline and drug regimen for the behavioral procedure (Extended Fig. 2-1). ***B***, Summary graph showing that morphine (50 ng, i.c.) injected directly into the PVT produced CPP [saline, *n* = 12 male mice; morphine (50 ng), *n* = 7 male mice; morphine (500 ng), *n* = 7 male mice; morphine (5 µg), *n* = 8 male mice; Extended Fig. 2-2 and Table 2-3). ***C***, Summary graph showing that coadministration of CTAP (1 ng) with morphine (50 ng) in the PVT blocked CPP [saline, *n* = 8 male mice; morphine (50 ng), *n* = 8 male mice; Mor (50 ng) + CTAP, *n* = 8 male mice]. ***D***, Summary graph showing activity counts during conditioning. a = unpaired chamber, b = paired chamber (saline, *n* = 4 male mice; 50 ng morphine, *n* = 5 male mice; 500 ng morphine, *n* = 6 male mice; 5 µg morphine, *n* = 7 male mice). ***E***, Summary graph showing activity counts before conditioning and 24 h after the last conditioning day [saline, *n* = 10 male mice; morphine (50 ng), *n* = 7 male mice; morphine (500 ng), *n* = 7 male mice; morphine (5 µg), *n* = 7 male mice]. *p < 0.05.

**Figure 3.**
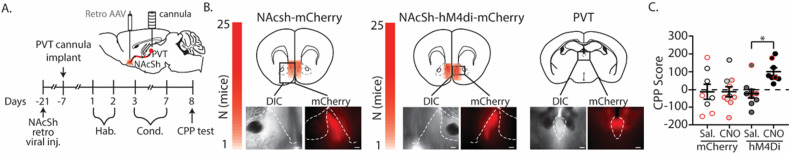
Inhibition of PVT projections to the NAcSh evokes CPP. ***A***, Experimental timeline and drug regimen for the behavioral procedure (Extended Fig. 3-1). PVT cannulated mice were injected with retrograde-mCherry or retrograde hM4di in the NAcSh (Extended Fig. 3-2). ***B***, Summary demonstrating viral injection placements in retrograde-mCherry and retrograde-hM4di–expressing mice. (Right) A representative image of viral expression in the PVT 6 weeks following viral injection of retrograde viral constructs in the NAcSh. Scale bar: 200 µm. ***C***, Summary graph showing the CPP score in mice conditioned with saline (equal volume) or CNO (3 µm) injected directly into the PVT [mCherry-sal, *n* = 4/4 (male/female); mCherry-CNO, *n* = 10 (male/female); hM4di-sal, *n* = 5/4 (male/female); hM4di-CNO, *n* = 6/2 (male/female); Extended Tables 3-3 and 3-4]. Red circles correspond to female mice. *p < 0.05.

**Figure 4.**
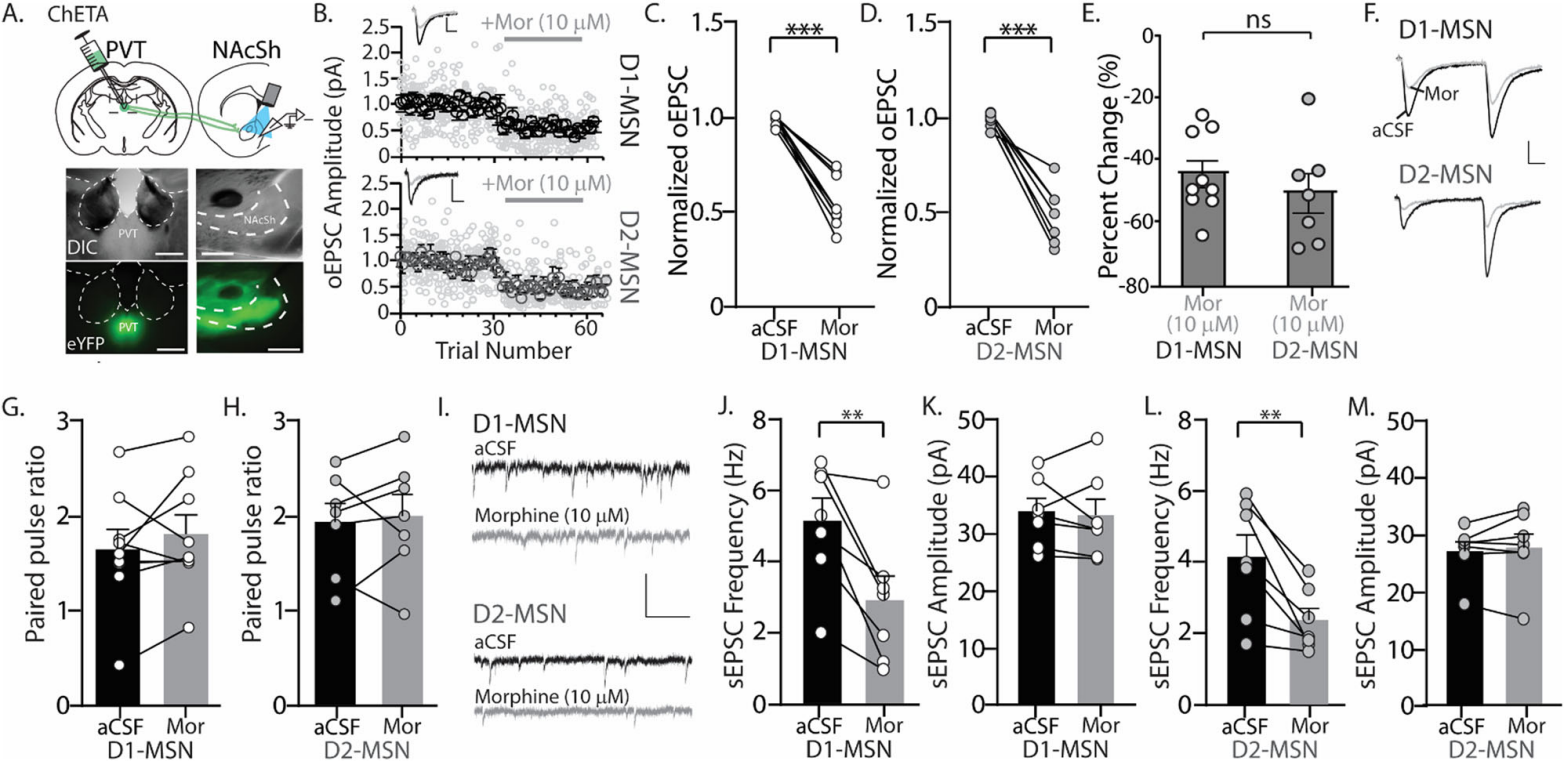
Bath application of morphine reduces EPSCs at PVT-to-D1-MSN and PVT-to-D2-MSN synapses in *Drd1a*-tdTomato mice. ***A***, (Top) Illustration showing the injection site of the channelrhodopsin mutant, ChETA, in the PVT and recording site in the NAcSh. (Bottom) DIC and fluorescent images showing that ChETA injection in the PVT evoked ChETA expression in the PVT and nucleus accumbens. Scale bar: 500 µm. ***B***, Graph showing the amplitude of optically evoked currents at PVT-to-D1-MSN (top) and PVT-to-D2-MSN synapses before and after bath application of morphine (10 µm). The dark circles represent the average, and the light gray circles show all data points from recorded neurons. Representative traces showing optically evoked paired-pulse EPSCs at both PVT-to-D1-MSN and PVT-to-D2-MSN synapses in the absence (black trace) and presence (gray trace) of bath-perfused morphine (10 µm). Scale bars: 50 pA, 10 ms. ***C***, Summary graph showing the normalized oEPSCs at PVT-to-D1-MSN (9 neurons/4 male mice) and (***D***) PVT-to-D2-MSN synapses (7 neurons/3 male mice) following bath application of morphine (10 µm). The circles represent individual MSNs. ***E***, Summary graph showing the percent change at PVT-to-D1-MSN and PVT-to-D2-MSN synapses following bath application of morphine (10 µm). ***F***, Representative traces showing optically evoked paired-pulse EPSCs at both PVT-to-D1-MSN and PVT-to-D2-MSN synapses in the absence (black trace) and presence (gray trace) of bath-perfused morphine (10 µm). Scale bars: 50 pA, 10 ms. ***G***, Summary graph showing the paired-pulse ratio at PVT-to-D1-MSN synapses before and after morphine bath application (10 µm; 9 neurons from 4 male mice). ***H***, Summary graph showing the paired-pulse ratio at PVT-to-D2-MSN synapses before and after morphine bath application (10 µm; 7 neurons from 3 male mice). ***I***, Representative traces showing sEPSCs recorded from D1-MSN and D2-MSN in the absence (black trace) and presence (gray trace) of bath-perfused morphine (10 µm). Scale bars: 50 pA, 200 ms. ***J***, Summary graph showing the sEPSC frequency and (***K***) amplitude on D1-MSNs in the absence and presence of morphine (7 neurons from 4 male mice). ***L***, Summary graph showing the sEPSC frequency and (***M***) amplitude on D2-MSNs in the absence and presence of morphine (7 neurons from 3 male mice). **p < 0.01, *** *p* < 0.001.

**Figure 5.**
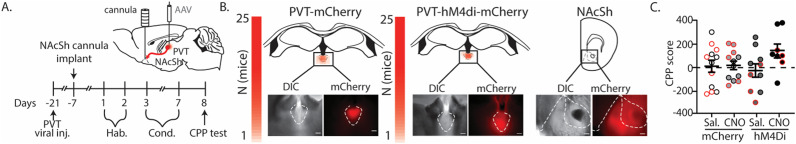
Inhibition of PVT-to-NAcSh synaptic transmission does not evoke CPP. ***A***, Experimental timeline and drug regimen for the behavioral procedure (Extended Fig. 5-1). NAcSh cannulated mice were injected with mCherry or hM4di in the PVT. ***B***, Summary demonstrating viral injection placements in mCherry- and hM4di-expressing mice (Extended Fig. 5-2). (Right) A representative image of viral expression in the NAcSh 6 weeks following viral injection of viral constructs in the PVT. Scale bar: 200 µm. ***C***, Summary graph showing the CPP score in mice conditioned with saline (equal volume) or CNO (3 µm) injected directly into the NAcSh [mCherry-sal, *n* = 6/6 (male/female); mCherry-CNO, *n* = 6/6 (male/female); hM4di-sal, *n* = 6/4 (male/female); hM4di-CNO, *n* = 5/3 (male/female); Extended Tables 5-3 and 5-4]. Red circles correspond to female mice.

All experiments were done in accordance with procedures approved by the Pennsylvania State University College of Medicine Institutional Animal Care and Use Committee. Total animal numbers were determined by power analyses [an effect size = 0.5, power = 0.80, and type 1 error (*α*) = 0.05]. This investigation was not designed to identify sex differences. Therefore, the power analysis did not take the number of males and females for each experiment into account. All mice (*N* = 149 males = 114; females = 35) used in this study were aged 8–12 weeks at the time of recording. Recordings investigating the effects of bath-perfused morphine from retrogradely labeled PVT neurons were conducted in naive male and female C57BL/6 mice (*n* = 7; Fig. 1). Cell-type–specific D1-MSN and D2-MSN recordings investigating the effects of bath-perfused morphine were made using naive male and female B6 Cg-Tg (*Drd1a*-tdTomato) line 6 Calak/J hemizygous mice (JAX stock #16204; *n* = 4; Fig. 4). Given that *Drd1a*-tdTomato transgenic mice have fluorescently labeled D1-MSNs, D2-MSNs were identified based on the lack of fluorescence, cell size, and electrophysiological characteristics, including capacitance and membrane resistance, as previously published (Graziane et al., 2016; McDevitt et al., 2019). Behavioral experiments were performed with male and female C57BL/6 mice, including experiments where morphine was injected directly into the PVT (*N* = 58; Fig. 2) and experiments implementing designer receptors exclusively activated by designer drugs [DREADDs; Fig. 3 (*N* = 35) and Fig. 5 (*N* = 43)]. Mice were singly housed and maintained on a regular 12 h light/dark cycle (lights on 07:00, lights off 19:00) with ad libitum food and water. Random placement of home cages within the housing and behavioral rooms was employed for electrophysiological and behavioral experiments to control any environmental factors (e.g., room lighting and vibrations; Steel et al., 2021). A total of 33 mice were excluded from this study due to misplaced cannula (*N* = 2), misplaced or lack of viral expression (Fig. 3; *N* = 12; Fig. 5; *N* = 17), and/or surgical complications (*N* = 2).

### Drugs

(−)-Morphine sulfate pentahydrate (diluted in saline) and clozapine *N*-oxide (CNO; diluted in saline with 0.1% DMSO) were provided by the National Institute on Drug Abuse Drug Supply Program. Picrotoxin was purchased from Sigma-Aldrich (catalog #P1675). The somatostatin analog D-Phe-Cys-Tyr-D-Trp-Arg-Thr-Pen-Thr-NH2 (CTAP) was purchased from Tocris Bioscience (catalog #1560).

### Stereotaxic surgery

Anesthesia was induced and maintained with isoflurane at 1.5%. The animal was placed in a stereotaxic frame (Stoelting), and craniotomies were performed via microdrill. Injections were carried out via a 33-gauge beveled-tip needle (World Precision Instruments) connected to a 5 µl Hamilton syringe on a micro pump (Harvard Apparatus) at an infusion rate of 200 nl/min. Following injection, the needle was left in situ for 5–10 min to allow for virus/tracer diffusion and then slowly retracted to limit backflow. For retrograde labeling experiments, 500 nl of cholera toxin subunit B (CTB) conjugated to Alexa Fluor 488 (Invitrogen; catalog #C-22841) diluted in sterile saline was injected into the medial NAcSh [from the bregma: anteroposterior (AP), +1.70 mm; mediolateral (ML), ±0.60 mm; dorsoventral (DV), −4.50 mm]. Mice were used for electrophysiology experiments 3–4 d postinjection. For optogenetic experiments, 200 nl of virus AAV5 (adenoassociated virus)-CaMKIIa-ChETA (E123T/H134R)-eYFP (Addgene; catalog #100050; titer, 2.4 × 10^13^ GC/ml) was injected into the midline PVT (from the bregma: AP, −1.60 mm; ML, ±0.00 mm; DV, −3.30 mm). Midline PVT was targeted as we have shown through electrophysiology approaches that morphine alters the excitability of these neurons (McDevitt and Graziane, 2019) and that the midline PVT projects to the NAcSh (Fig. 1). Mice were used for electrophysiology experiments 4–5 weeks postinjection to allow for virus expression. Mice used for DREADD manipulations were injected with an AAV2-retro–engineered serotype (Tervo et al., 2016), pAAV-hSyn-hM4D(Gi)-mCherry (AAVrg) (Addgene; catalog #50475-AAVrg; titer, 2.4 × 10^13^), or pAAV-hSyn-mCherry (AAVrg) (Addgene; catalog #114472-AAVrg; titer, 2.4 × 10^13^) in the NAcSh. For DREADD injections in the PVT, pAAV-hSyn-mCherry (Addgene; catalog #114472-AAV5; titer, 8.6 × 10^12^) and pAAV-hSyn-hM4D(Gi)-mCherry (Addgene; catalog #50475-AAV5; titer, 8.6 × 10^12^) were used. Behavioral experiments occurred 4–5 weeks postinjection to allow for viral expression. Viral vectors were a gift from Karl Deisseroth or Bryan Roth.

#### PVT cannula procedure

PVT cannulas were fabricated based on a previously published procedure (Kazanskaya et al., 2020) using a fabricated jig (https://github.com/omn0mn0m/PVT-Cannula-Jig) or purchased [catalog #C315GA length 2.3 µm; Protech International Inc. (Boerne, Texas)]. One week prior to CPP training, mice were implanted with a 26-gauge guide cannula targeting the PVT (from the bregma: AP, −1.60 mm; ML, 0.00 mm; DV, −2.30 mm). On CPP conditioning day, in the absence of anesthesia, 200 nl of saline, morphine (50 ng), or Mor + CTAP (1 ng) was injected through the guide cannula using a 33-gauge needle extending 1 mm past the guide cannula into the PVT (from the bregma: AP, −1.60 mm; ML, 0.00 mm; DV, −3.30 mm; catalog #C315IA 33-gauge internal cannula injector 3.3 mm length; Protech International Inc. (Boerne, Texas)] at an infusion rate of 300 nl/min. In addition, 1 ng of CTAP was used based on a previous publication (Simmons and Self, 2009). For DREADD experiments, CNO (3 µm, 300 nl) was injected into the PVT at an infusion rate of 300 nl/min as we have shown that this concentration of CNO is sufficient to activate hM4D(Gi)-DREADDs (McKendrick et al., 2022) and others have shown that 3 µm CNO is sufficient to activate hM4D(Gi)-DREADDs in the PVT (Keyes et al., 2020).

#### NAcSh cannula procedure

NAcSh cannulas were purchased from PlasticsOne (catalog #8IC235GS512S; length 3.5). One week prior to CPP training, mice were implanted with a 26-gauge guide cannula targeting the NAcSh (from the bregma: AP, +1.70 mm; ML, 0.60 mm; DV, −3.50 mm). On CPP conditioning, in the absence of anesthesia, CNO (3 µm, 500 nl) was injected through the guide cannula using a 33-gauge needle extending 1 mm past the guide cannula into the NAcSh (catalog #8IC235IS5SPC, 33-gauge internal cannula injector 3.6 mm length; PlasticsOne) at an infusion rate of 300 nl/min.

#### Cannula placement

Cannula placements were checked (Figs. 2, 3, 5) by injecting 5 mM Evans blue (MP Biomedicals; catalog #15108) through the guide cannula. Infusion rates for dye injection were consistent with those used for drug injection during experimentation. Brain slices were then prepared on a Leica VT1200S vibratome to identify the location and diffusion of dye through the tissue.

### Noncontingent CPP

CPP was performed as previously described (McDevitt and Graziane, 2019; McKendrick et al., 2020a,b). Briefly, CPP chambers (Med Associates) were in the mouse housing room and consisted of three distinct compartments separated by manual guillotine-style doors. Each compartment had distinct contextual characteristics: the middle (neutral) compartment (7.2 cm × 12.7 cm × 12.7 cm) had gray walls and gray plastic floor, while the choice compartments (16.8 cm × 12.7 cm × 12.7 cm, each) had either white walls and stainless-steel mesh floor or black walls and stainless-steel grid floor. All compartments were illuminated with the same dim light intensity during use. Immediately following use the entire preference chamber was cleaned thoroughly with a scent-free soap solution. Mouse locations, activity counts, and time spent in each compartment were collected via automated data collection software (Med Associates) via infrared photobeam strips lining each compartment.

#### Habituation

Mice were placed in the center compartment with free access to all three compartments for 20 min once a day for 2 d. Time spent (seconds) in each compartment was recorded.

#### Conditioning (acquisition phase)

Twenty-four hours after habituation, mice received 5 d conditioning training. Morphine-paired compartments were assigned based on the least preferred side (Tzschentke, 2007) calculated by averaging time spent in each compartment over the 2 habituation days (Extended Fig. 2-1, 3-1, and 5-1). Similar to conditioning studies with alcohol (Gremel et al., 2006), we find that C57BL/6 mice will reliably develop morphine CPP by pairing morphine with the least preferred chamber (McDevitt and Graziane, 2019; McKendrick et al., 2020a,b). In this assay, randomly assigning compartments may underestimate the CPP score, especially when animals are conditioned with an experimental treatment in their preferred chamber. Therefore, a biased design provides a more accurate measurement for these reward-related assays.

At the start of conditioning sessions (09:00), mice received an injection of saline directly in the PVT and were placed into the most preferred compartment (unpaired chamber) for 20 min. After this training session, mice were placed back in their home cage. 4–6 h later (13:00–15:00), mice received an injection of saline (control group), morphine (50 ng, 500 ng, or 5 µg), or Mor (50 ng) + CTAP (1 ng) in the PVT (Fig. 2). The dose range of morphine was determined based on previous studies investigating intracranial effects of morphine (Sharpe et al., 1974; Bozarth and Wise, 1981; David and Cazala, 1994). Experiments investigating the effects of hM4D(Gi) DREADDs consisted of saline or CNO (3 µm) injections directly in the PVT (Fig. 3) or NAcSh (Fig. 5) followed by placement in the most (saline) or least preferred compartment (CNO; paired chamber) for 20 min (Koo et al., 2014; Graziane et al., 2016).

#### Postconditioning (expression phase)

One day after the last conditioning day (11:00), mice were placed in the three-compartment chamber and allowed to move freely for 20 min. CPP scores for both the paired compartments were calculated as time spent on the respective side on test day minus the average time spent on the same side during preconditioning (Bohn et al., 2003). Activity counts are defined as any beam break within a current zone, inclusive of grooming, rearing, and lateral movements.

#### Acute brain slice preparation

Mice were deeply anesthetized with isoflurane and cardiac perfused with an ice-cold *N*-methyl-D-glucamine (NMDG)-based cutting solution containing (in mM) 135 NMDG, 1 KCl, 1.2 KH_2_PO_4_, 0.5 CaCl_2_, 1.5 MgCl_2_, 20 choline-HCO_3_, and 11 glucose, saturated with 95%O_2_/5%CO_2_, adjusted to a pH of 7.4 with HCl, and with osmolality adjusted to 305 mmol/kg. Following perfusion, mice were decapitated, and brains were rapidly removed. In addition, 250 µm coronal brain slices containing the NAcSh or PVT were prepared via a Leica VT1200S vibratome in a 4°C NMDG cutting solution. Following cutting, slices were allowed to recover in artificial cerebrospinal fluid (aCSF) containing (in mM) 119 NaCl, 2.5 KCl, 2.5 CaCl_2_, 1.3 MgCl_2_, 1 NaH_2_PO_4_, 26.2 NaHCO_3_, and 11 glucose, with osmolality of 290 mmol/kg, at 31°C for 30 min followed by 30 min at 20–22°C prior to recording. Slices were kept at 20–22°C for the rest of the recording day.

### Whole-cell electrophysiology

All recordings were made from either the medial NAcSh between bregma +1.70 mm and +0.86 mm or the middle PVT between bregma −1.22 and −1.70. Slices were transferred to a recording chamber, and neurons were visualized using infrared differential interference contrast (DIC) microscopy. During recording, slices were superfused with aCSF at 30°C. Where indicated, brain slices were perfused with morphine (10 µm) and/or CTAP (1 µm), the µ-opioid receptor antagonist. Morphine and CTAP concentrations were selected based on previously published findings (Connor et al., 1996; Connor and Christie, 1998; Margas et al., 2010; Mahmoud et al., 2011; Levitt et al., 2015).

For intrinsic membrane excitability (IME) measurements and rheobase measurements, recording electrodes [3–5 MΩ; borosilicate glass capillaries (World Precision Instruments #1B150F-4)] were pulled on a horizontal puller from Sutter Instruments (Model P-97) and filled with a potassium-based internal solution containing (in mM) 130 KMeSO_3_, 10 KCl, 10 HEPES, 0.4 EGTA, 2 MgCl_2_-6H_2_0, 3 Mg-ATP, and 0.5 Na-GTP, with pH 7.2–7.4 and osmolality = 290 mmol/kg (Wescor Vapro Model 5600, ELITechGroup).

Resting membrane potential (RMP) was recorded immediately following break-in. For IME experiments, we employed a commonly used current step protocol (Ishikawa et al., 2009; McDevitt et al., 2019; McDevitt and Graziane, 2019; Roselli et al., 2020). In this study, the IME and rheobase protocols were conducted at unadjusted resting membrane potentials. Our current step protocol, consisting of 1 s steps ranging from −100 to 100 pA in 50 pA increments, was carried out with a 20 s intrasweep interval. The number of action potentials observed at each current step was recorded. For rheobase experiments, a 2 s consistent-slope current injection ramp with a maximal current of 400 pA was performed, as previously described (Pati et al., 2020). The rheobase was defined as the minimal current needed to elicit an action potential.

For recording optically evoked excitatory postsynaptic currents (oEPSCs), in the NAcSh, recording electrodes (3–5 MΩ; borosilicate glass capillaries, World Precision Instruments #1B150F-4) were pulled on a horizontal puller from Sutter Instruments and filled with a cesium-based internal solution containing (in mM) 135 CsMeSO_3_, 5 CsCl, 5 TEA-Cl, 0.4 EGTA (Cs), 20 HEPES, 2.5 Mg-ATP, 0.25 Na-GTP, and 1 QX-314 (Br), with pH 7.2–7.4 and osmolality = 290 mmol/kg. To evoke optical excitatory postsynaptic currents (oEPSCs), PVT presynaptic afferents were stimulated at 0.1 Hz by flashing 470 nm light (0.1–0.3 ms duration) through the light path of the microscope using an LED-based light source (X-Cite 120LED Boost, Excelitas Technologies). Following cell stabilization after break-in, a stable baseline of 30–50 AMPA-mediated oEPSCs was obtained at −70 mV command voltage. The AMPA-mediated peak amplitude was determined by measuring the amplitude of oEPSCs at −70 mV command voltage. Paired-pulse recordings were performed with two optically evoked stimuli separated by 50 ms.

All recordings were performed using either an Axon Multiclamp 700B amplifier or Sutter Double IPA, filtered at 2–3 kHz and digitized at 20 kHz. Series resistance was typically 10–25 MΩ, left uncompensated, and monitored throughout. For all voltage-clamp recordings, cells with a series resistance variation >20% were discarded from the analysis. For all current-clamp recordings, cells with a bridge balance that varied >20% during the start and end of recordings were discarded from analysis. Percent change was calculated as [(final value − baseline value)/absolute baseline value) × 100.

### Statistical analysis

All results are shown as mean ± SEM. Data sets were tested for normality and equality of variances, and the appropriate statistical measures were performed. Statistical significance was assessed in GraphPad Prism software using a paired Student's *t* test and one- or two-way ANOVA with Bonferroni's correction for multiple comparisons to identify differences as specified. *F*-values for two-way ANOVA statistical comparisons represent interactions between variables unless otherwise stated. Two-tail tests were performed for all studies. The effect sizes for group comparisons were quantified using Cohen's *d*, which expresses the magnitude of the difference between group means in terms of standard deviations (Sullivan and Feinn, 2012).

## Results

### Bath application of morphine reduces excitability in PVT neurons that project to the NAcSh

We first investigated whether morphine directly influenced the neuronal excitability of PVT neurons that project to the NAcSh. To do this, we retrogradely labeled PVT neurons by injecting CTB-488 into the NAcSh. This permitted CTB to be taken up into the cytoplasm of PVT afferents that innervate the NAcSh, resulting in fluorescently labeled PVT neurons 3–4 d later (Fig. 1*A*). In addition, 3–4 d after CTB injections, brain slices containing the PVT were prepared for whole-cell electrophysiology recordings. Fluorescently labeled PVT neurons were current-clamped and recorded in the absence (aCSF) and presence of bath-perfused morphine (10 µm). We observed that upon bath application of morphine, the RMP of PVT neurons that project to the NAcSh was significantly hyperpolarized (*t*_(7) _= 3.03; *p* = 0.0192; paired Student's *t* test; Fig. 1*B*). To investigate whether this effect was mediated by activation of µ-opioid receptors, we bath-applied morphine in the presence of CTAP (1 µm), the selective µ-opioid receptor antagonist, and observed no significant change in the RMP (*t*_(9) _= 1.338; *p* = 0.2138; paired Student's *t* test; Fig. 1*C*). Additionally, we found a significant decrease in the percent change of the RMP following bath application of morphine in absence compared with the presence of CTAP (*t*_(16) _= 3.475; *p* = 0.0031; Student's *t* test; Fig. 1*D*), suggesting that morphine-induced hyperpolarization of PVT neurons is mediated through µ-opioid receptors.

Next, we investigated the effects of morphine on the rheobase, the minimum current required to evoke an action potential, in PVT neurons that project to the NAcSh. Increases in the rheobase suggest an increased threshold for neuronal excitability, indicating that the neuron has become less responsive to current input. Conversely, decreases in the rheobase suggest a lowered threshold for neuronal excitability, indicating that the neuron has become more responsive to a stimulus. We found that in the presence of morphine, the rheobase was significantly increased (*t*_(7) _= 3.557; *p* = 0.0093; paired Student's *t* test; Fig. 1*E*). Furthermore, we observed that the effects of morphine were mediated through µ-opioid receptors as CTAP prevented morphine-induced changes (*t*_(9) _= 0.6186; *p* = 0.5515; paired Student's *t* test; Fig. 1*F*) and blocked morphine-induced increases in the rheobase (*t*_(16) _= 2.521; *p* = 0.0227; Student's *t* test; Fig. 1*G*).

Lastly, we measured the IME in PVT neurons that project to the NAcSh in the presence of morphine. We found that the IME was significantly decreased (*F_(_*_10,120) _= 7.840; *p* < 0.0001; two-way repeated measures ANOVA with Bonferroni’s posttest; Fig. 1*H*), and this morphine-induced effect was blocked by CTAP (80 pA: Mor vs Mor + CTAP, *p* = 0.0194; aCSF vs Mor + CTAP, *p *> 0.9999; 100 pA: Mor vs Mor + CTAP, *p* = 0.0196; aCSF vs Mor + CTAP, *p *> 0.9999; Bonferroni’s posttest; Fig. 1*H*). Overall, our observed morphine-induced inhibition of PVT neurons is consistent with what has been observed with DAMGO, an MOR agonist (Brunton and Charpak, 1998).

### µ-Opioid receptor activation in PVT neurons that project to the NAcSh is sufficient to evoke opioid reward

Given the evidence that activation of PVT-to-NAc synaptic transmission evokes aversive behaviors (Bubser and Deutch, 1999; Zhu et al., 2016; Beas et al., 2018), we next investigated whether the inhibitory actions of morphine in the PVT were sufficient to evoke opioid reward. To test this, we performed CPP experiments, as CPP is reliably used to measure the rewarding properties of drugs (McKendrick and Graziane, 2020). Mice underwent CPP training consisting of two habituation days, followed by 5 d of conditioning, and CPP was measured on postconditioning day 1 (Fig. 2*A*). Saline (equal volume, i.c.) or varying doses of morphine (50 ng, 500 ng, 5 µg, i.c.) were injected directly into the PVT during conditioning sessions with cannula placements verified after experimentation (Extended Fig. 2-2). We found that mice conditioned with morphine (50 ng, i.c.) displayed a preference for the morphine-paired chamber [*F*_(3,30) _= 3.051, *p *= 0.0436; one-way ANOVA with Bonferroni’s posttest; saline vs morphine (50 ng), *p *= 0.0471, Cohen's *d* = 1.92; saline vs morphine (500 ng), *p *= 0.6117, Cohen's *d* = 0.829; saline vs morphine (5 µg), *p *= 0.3706, Cohen's *d* = 0.857; morphine (50 ng) vs morphine (500 ng), *p *> 0.9999, Cohen's *d* = 0.583; morphine (50 ng) vs morphine (5 µg), *p *> 0.9999, Cohen's *d* = 0.451; morphine (500 ng) vs morphine (5 µg), *p *> 0.9999, Cohen's *d* = 0.065; Fig. 2*B* and Extended Table 2-3]. To test whether morphine (50 ng)-induced CPP was caused by µ-opioid receptor activation, we repeated CPP experiments by conditioning separate groups of mice with saline (i.c. in PVT) or 50 ng of morphine in the absence and presence of CTAP (1 ng, i.c. in PVT). The results show that in the presence of CTAP, morphine (50 ng i.c. in the PVT)-induced CPP was blocked (*F*_(2,21) _= 6.149, *p *= 0.0079; one-way ANOVA with Bonferroni’s posttest; saline vs Mor, *p *= 0.0110; Mor vs Mor + CTAP, *p *= 0.0376; Bonferroni’s posttest; Fig. 2*C*) suggesting that morphine injected into the PVT evokes CPP through µ-opioid receptor activation.

10.1523/ENEURO.0524-23.2024.f2-1 Figure 2-1 Summary graph of the CPP data in Figure 2 showing the average time spent on the drug-paired chamber (least preferred) and control-paired chamber (most preferred) during two habituation sessions (saline: n = 20; morphine (50 ng): n = 15; morphine (500 ng): n = 7; morphine (5 μg): n = 8; morphine (50 ng) + CTAP: n = 8). Download Figure 2-1, TIF file.

10.1523/ENEURO.0524-23.2024.f2-2 Figure 2-2 Summary of PVT cannula placements for mice undergoing CPP with direct morphine injections into the PVT. The figure represents Evans Blue staining of individual animals. Intensity of the stain is based on the number of animals with an N of 1 corresponding to low intensity and N of 20 the maximum intensity. Download Figure 2-2, TIF file.

10.1523/ENEURO.0524-23.2024.t2-3 Table 2-3 Extended data table providing effect size comparisons for CPP data supporting Figure 2. Download Table 2-3, DOC file.

In addition to measuring CPP, we monitored locomotor activity following morphine delivery directly in the PVT as significant increases in locomotor activity are observed following systemic injection of morphine in C57BL/6 mice (Graziane et al., 2016; McKendrick et al., 2020a,b). To do this, we measured activity counts defined as any beam break within a current zone, inclusive of grooming, rearing, and lateral movements. Activity counts were measured during each conditioning session (conditioning days 1–5) in the absence (unpaired chamber) or presence (paired chamber) of morphine injections in the PVT. Our results show that morphine at all doses administered had no effect on the activity counts during conditioning sessions (*F*_(27,162) _= 1.547, *p *= 0.0519; two-way repeated measures ANOVA; Fig. 2*D*).

Lastly, systemic injections of escalating doses of morphine have been shown to reduce locomotor activity 24 h after the last exposure, which is associated with spontaneous opioid withdrawal (Madayag et al., 2019; McDevitt et al., 2021). Because of this, we compared the activity of mice before conditioning sessions (habituation) with the activity of mice after morphine exposure (test). If mice were undergoing spontaneous opioid withdrawal, a significant decrease in locomotor activity would be expected to occur during the postconditioning test compared with locomotor activity observed during habituation. Our results show that morphine (50 ng, 500 ng, or 5 µg) injected directly into the PVT did not alter activity counts 24 h after the last conditioning day (*F*_(4,26) _= 0.7325, *p *= 0.5758; two-way repeated measures ANOVA; Fig. 2*E*).

Morphine inhibits neuronal activity through G_i/o_-protein–coupled receptor signaling and subsequent opening of potassium channels (Koski and Klee, 1981; Williams et al., 1982; Hsia et al., 1984; Schroeder et al., 1991; Johnson et al., 1994; Moises et al., 1994; Schneider et al., 1998). Our results show that morphine decreases neuronal excitability of PVT neurons that project to the NAcSh (Fig. 1). Additionally, our results show that morphine injected directly into the PVT is associated with the acquisition of opioid context associations (Fig. 2). Therefore, we next investigated whether inhibiting PVT neurons that project to the NAcSh, using chemogenetic approaches (Urban and Roth, 2015), mimicked the rewarding effects of morphine. For these experiments, we employed a retrograde hM4Di-DREADD, which when stimulated by CNO, activates G-protein inwardly rectifying potassium channels resulting in hyperpolarization and attenuation of neuronal activity (Armbruster et al., 2007). The retrograde hM4Di-DREADD was injected bilaterally into the NAcSh, permitting retrograde transport of the hM4Di-DREADD to the PVT via PVT afferents in the NAcSh (Fig. 3*A,B*). A cannula was implanted in the PVT for local delivery of CNO, and cannula placements were verified after experimentation (Extended Fig. 3-2). Mice underwent CPP training consisting of two habituation days, followed by 5 d of conditioning where saline (equal volume, i.c.) or CNO (3 µm, i.c.) was injected directly into the PVT and CPP was measured on postconditioning day 1. We found that hM4Di-expressing mice conditioned with CNO (3 µm, i.c.) displayed CPP for the CNO-paired chamber compared with control mice (*F*_(1,31) _= 4.370, *p *= 0.045; two-way ANOVA with Bonferroni’s posttest; mCherry(sal) vs mCherry(CNO), *p* > 0.9999, Cohen's *d* = 0.001; mCherry(sal) vs hM4Di(sal), *p* > 0.9999, Cohen's *d* = 0.110; mCherry(sal) vs hM4Di(CNO), *p* = 0.0853, Cohen's *d* = 1.23; mCherry(CNO) vs hM4Di(sal), *p* > 0.9999, Cohen's *d* = 0.132; mCherry(CNO) vs hM4Di(CNO), *p* = 0.0612, Cohen's *d* = 1.47; hM4Di(sal) vs hM4Di(CNO), *p* = 0.0384, Cohen's *d* = 1.84; Fig. 3*C* and Extended Tables 3-3 and 3-4).

10.1523/ENEURO.0524-23.2024.f3-1 Figure 3-1 Extended data summary of Figure 3 CPP data showing the average time spent on the drug-paired chamber (least preferred) and control-paired chamber (most preferred) during two habituation sessions (mCherry-sal: n = 8; mCherry-CNO: n = 10; hM4Di-sal: n = 9; hM4Di-CNO: n = 8). Download Figure 3-1, TIF file.

10.1523/ENEURO.0524-23.2024.f3-2 Figure 3-2 Extended data summary of PVT cannula placements for mice undergoing CPP with direct CNO injections into the PVT. The figure represents Evans Blue staining of individual animals. Intensity of the stain is based on the number of animals with an N of 1 corresponding to low intensity and N of 20 the maximum intensity. Download Figure 3-2, TIF file.

10.1523/ENEURO.0524-23.2024.t3-3 Table 3-3 Extended data table providing effect size comparisons for CPP data supporting Figure 3. Download Table 3-3, DOC file.

10.1523/ENEURO.0524-23.2024.t3-4 Table 3-4 Extended data table supporting Figure 3 showing main effects of virus (mCherry and hM4Di) and treatment (sal and CNO). Download Table 3-4, DOC file.

### Bath application of morphine reduces excitatory postsynaptic currents (EPSCs) at PVT-to-D1-MSN and PVT-to-D2-MSN synapses

Thus far, our results suggest that inhibition of PVT neurons that project to the NAcSh is an important pathway involved in mediating the acquisition of reward-related context associations. However, PVT axons are known to bifurcate with one neuron sending axons to multiple brain regions (Li et al., 2021; Viena et al., 2022). Therefore, we next investigated the inhibitory effects of morphine directly on PVT-to-NAcSh synapses using brain slice electrophysiology approaches. For these experiments, the channelrhodopsin mutant, ChETA, was injected into the PVT of *Drd1a*-tdTomato mice, permitting ChETA expression on PVT afferents in the NAcSh (Fig. 4*A*). *Drd1a*-tdTomato mice were used to assess cell-type–specific synaptic alterations in the NAcSh (Ade et al., 2011) through investigations on D1-MSNs and D2-MSNs, the main output neurons of the NAcSh (McDevitt and Graziane, 2018). Whole-cell voltage-clamp recordings were performed on D1-MSNs or D2-MSNs, and oEPSCs were recorded at PVT-to-D1-MSN or PVT-to-D2-MSN synapses. The magnitude of morphine-induced decrease in oEPSCs was determined as the mean oEPSC peak amplitude 2 min just before morphine application compared with mean oEPSC peak amplitude during the final 2 min period of morphine application, corresponding to trials 48–60 (Fig. 4*B*). Our results show that bath application of morphine (10 µm) significantly reduced the optically evoked current at both PVT-to-D1-MSN (*t*_(16) _= 9.546; *p* < 0.001; paired Student's *t* test; Fig. 4*B,C*) and PVT-to-D2-MSN synapses (*t*_(12) _= 8.539; *p* < 0.001; paired Student's *t* test; Fig. 4*B,D*). We also observed that this morphine-induced decrease was not significantly different at PVT-to-D1-MSN versus PVT-to-D2-MSN synapses (*t*_(14) _= 0.8335; *p* = 0.4186; paired Student's *t* test; Fig. 4*E*), suggesting that morphine inhibits PVT neurotransmission similarly at both D1-MSNs and D2-MSNs.

To identify whether morphine inhibits PVT-to-MSN synaptic transmission through pre or postsynaptic mechanisms, we measured the paired-pulse ratio, whereby the peak amplitude of the second pulse was divided by the peak amplitude of the first pulse. An increase in the paired-pulse ratio is associated with presynaptic inhibition, a decrease in the ratio is associated with presynaptic potentiation, and no change in the paired-pulse ratio reflects a postsynaptic modification (Creager et al., 1980; Harris and Cotman, 1983; Brundege and Williams, 2002). We observed no significant change in the paired-pulse ratio during bath application of morphine at PVT-to-D1-MSN (*t*_(8) _= 1.204; *p* = 0.2629; paired Student's *t* test; Fig. 4*F,G*) or PVT-to-D2-MSN synapses (*t*_(6) _= 0.445; *p* = 0.6720; paired Student's *t* test; Fig. 4*F,H*). However, in analyzing spontaneous EPSCs (sEPSCs), which are inclusive of all glutamatergic inputs in the NAcSh, we found that morphine significantly decreased sEPSC frequency with no effect on sEPSC amplitude when recording from D1-MSNs (frequency, *t*_(6) _= 4.055; *p* = 0.0067; paired Student's *t* test; amplitude, *t*_(6) _= 0.4487; *p* = 0.6694; paired Student's *t* test; Fig. 4*I,J,K*) or D2-MSNs (frequency, *t*_(6) _= 3.959; *p* = 0.0075; paired Student's *t* test; amplitude, *t*_(6) _= 0.7256; *p* = 0.4954; paired Student's *t* test; Fig. 4*I,L,M*). Given that changes in sEPSC frequency are associated with presynaptic modifications and changes in sEPSC amplitude are associated with postsynaptic modifications, these results suggest that circuit-specific modifications account for morphine-induced inhibition in the NAcSh.

Our results suggest that morphine decreases synaptic transmission at PVT-to-NAcSh projecting neurons, but whether inhibiting PVT synaptic transmission in the NAcSh is involved in the acquisition of context associations remains unknown. Therefore, we next investigated whether direct inhibition of PVT to NAcSh synaptic transmission was sufficient to evoke reward-related context associations. For these experiments, hM4Di-DREADD was injected into the PVT (Fig. 5*A*). A guide cannula was implanted in the NAcSh for local delivery of CNO [verified after experimentation (Extended Fig. 5-2)], thus permitting hM4Di-DREADD activation on PVT presynaptic terminals in the NAcSh (Fig. 5*B*). Mice underwent CPP training consisting of two habituation days, followed by 5 d of conditioning where saline (equal volume, i.c.) or CNO (3 µm, i.c., 500 nl) was injected directly into the NAcSh during conditioning sessions, thereby inhibiting PVT synaptic transmission. CPP was measured on postconditioning day 1. We found that hM4Di-expressing mice conditioned with CNO (3 µm, i.c.) displayed no significant CPP for the CNO-paired chamber compared with control mice (*F*_(1,38) _= 2.400, *p *= 0.1297; two-way ANOVA; mCherry(sal) vs mCherry(CNO), *p* > 0.9999, Cohen's *d* = 0.050; mCherry(sal) vs hM4Di(sal), *p* > 0.9999, Cohen's *d* = 0.204; mCherry(sal) vs hM4Di(CNO), *p* = 0.5417, Cohen's *d* = 0.983; mCherry(CNO) vs hM4Di(sal), *p* > 0.9999, Cohen's *d* = 0.291; mCherry(CNO) vs hM4Di(CNO), *p* = 0.6626, Cohen's *d* = 1.17; hM4Di(sal) vs hM4Di(CNO), *p* = 0.2312, Cohen's *d* = 1.23; Fig. 5 and Extended Tables 5-3 and 5-4).

10.1523/ENEURO.0524-23.2024.f5-1 Figure 5-1 Extended data summary graph of Figure 5 CPP data showing the average time spent on the drug-paired chamber (least preferred) and control-paired chamber (most preferred) during two habituation sessions (mCherry-sal: n = 12; mCherry-CNO: n = 12; hM4Di-sal: n = 10; hM4Di-CNO: n = 8). Download Figure 5-1, TIF file.

10.1523/ENEURO.0524-23.2024.f5-2 Figure 5-2 Extended data summary of NAcSh cannula placements for mice undergoing CPP with direct CNO injections into the NAcSh. The figure represents Evans Blue staining of individual animals. Intensity of the stain is based on the number of animals with an N of 1 corresponding to low intensity and N of 25 the maximum intensity. Download Figure 5-2, TIF file.

10.1523/ENEURO.0524-23.2024.t5-3 Table 5-3 Extended data table providing effect size comparisons for CPP data supporting Figure 5. Download Table 5-3, DOC file.

10.1523/ENEURO.0524-23.2024.t5-4 Table 5-4 Extended data table supporting Figure 5 showing main effects of virus (mCherry and hM4Di) and treatment (sal and CNO). Download Table 5-4, DOC file.

Finally, we observed differences in the variability of data presented in Figures 3 and 5 among the experimental groups (hM4Di(CNO)-PVT vs hM4Di(CNO)-NAcSh). We conducted an analysis to explore the variance in the CPP scores between these two groups. We observed a significant difference in the variance of the CPP score between mice with inhibition of PVT neurons that project to the NAcSh (Fig. 3) versus mice with inhibition of PVT presynaptic terminals in the NAcSh (Fig. 5; *F*_(8,7) _= 7.631; *p* = 0.0146; unpaired Student's *t* test with an *F* test to compare variances).

## Discussion

Here, we show that morphine inhibits PVT neurons that project to the NAcSh and reduces synaptic transmission at both PVT-to-D1-MSN and PVT-to-D2-MSN synapses in the NAcSh. Additionally, we provide evidence that the PVT is necessary for the acquisition of morphine CPP as direct injections of morphine in the PVT resulted in place preference for the morphine-paired chamber.

Recent reports have shown that morphine and DAMGO reduce PVT neuron firing rates, that DAMGO hyperpolarizes amygdala-projecting PVT neurons, that DAMGO inhibits glutamatergic synaptic transmission at PVT to MSNs in the NAc, and that heroin reduces the activity of PVT-to-NAc neurons (Goedecke et al., 2019; Vollmer et al., 2022; Hou et al., 2023). Our results are consistent with these findings as we show that PVT neurons that project to the NAcSh express hyperpolarized resting membrane potentials, increases in rheobase, and decreases in the IME upon bath perfusion of morphine (Fig. 1). The hyperpolarizing effects of MOR agonists on PVT neurons is not surprising considering that MOR activation results in the activation of inwardly rectifying potassium channels (Zamponi and Snutch, 1998; Torrecilla et al., 2002, 2008), thus causing positively charged K^+^ ions to move from the intracellular to extracellular space, leading to hyperpolarization of the membrane potential.

Not only did morphine inhibit neuronal excitability of PVT neurons that projected to the NAcSh, but morphine also inhibited synaptic transmission at both PVT-to-D1-MSN and D2-MSN synapses in the NAcSh. However, more studies are required to elucidate the pre and/or postsynaptic mechanisms mediating this inhibitory effect. Given that MORs are expressed on PVT neurons, it would be expected that MOR activation on PVT terminals in the NAcSh would result in reduced presynaptic neurotransmitter release. This change in the presynaptic release can be measured using electrophysiological approaches like the paired-pulse ratio. During the bath application of morphine, we did not observe a significant change in the paired-pulse ratio at PVT-to-D1-MSN or PVT-to-D2-MSN synapses, as both the first and second pulse were inhibited similarly by morphine. However, when analyzing spontaneous excitatory postsynaptic currents (sEPSCs), inclusive of all glutamatergic inputs projecting to the NAcSh, we observed a significant decrease in sEPSC frequency in the presence of bath-applied morphine. Changes in sEPSC frequency support a presynaptic modification. In contrast, we did not observe a significant change in the sEPSC amplitude, which is commonly associated with postsynaptic modifications. The typical mechanisms of opioid receptor–mediated neurotransmission involve presynaptic modulation of neurotransmitter release or postsynaptic modulation of voltage-gated channels (Reeves et al., 2022). The internal solution used in our recordings included voltage-gated channel blockers, enabling us to isolate synaptically-mediated currents. Therefore, our results suggest that PVT-to-MSN synapses in the NAcSh possess a unique response to morphine that may include postsynaptic modulation of synaptically expressed receptors. In line with this, Zhu et al. (2016) demonstrated that chronic morphine treatment and in vivo optogenetic long-term depression did not affect the paired-pulse ratio of MSNs receiving PVT input.

Evidence suggests that activation of the PVT and its afferents in the central amygdala and nucleus accumbens leads to aversive behaviors (Penzo et al., 2015; Zhu et al., 2016; Zhou et al., 2022). Therefore, one may expect that inhibition of the PVT, via MOR activation, would result in reward-related behavioral phenotypes. Here, we show that the morphine-induced inhibition of PVT neurons is sufficient to generate the acquisition of CPP following direct injections of morphine into the PVT (Fig. 2). However, this effect was only observed with 50 ng (330 µm) of morphine and not with morphine injections of 500 ng (3.30 mM) or 5 µg (33.0 mM). Given that morphine is a weak agonist at κ-opioid receptors (*K*_i_ = ∼115 nM; Ben Haddou et al., 2014; Miyazaki et al., 2017), this may be due to off-target activation at κ-opioid receptors, which are expressed in the PVT (Le Merrer et al., 2009). Future experiments will look to see whether coinfusing these higher morphine doses with a κ-opioid receptor antagonist can “un-mask” CPP. Notably, we found that morphine injections directly into the PVT had no effect on locomotor activity, suggesting that the PVT is not associated with the locomotor sensitization often observed after systemic morphine injection in C57BL/6 mice (Walters et al., 2005; Contet et al., 2008; Guegan et al., 2016; Bulin et al., 2020; Leite Júnior et al., 2023). Locomotor sensitization is highly dependent on the context in which the drug is administered (Robinson and Berridge, 2003); thus, it is possible in another context we might observe a sensitized response to acute morphine injections in the PVT. However, similar results were obtained when PVT projections to the central amygdala were inhibited and no effect on locomotor sensitization evoked by systemic morphine injections was observed (Keyes et al., 2020). Additionally, this same study found that optical activation of PVT neurons that project to the central amygdala had no effect on locomotor activity (Keyes et al., 2020).

Our findings also show that chemogenetic inhibition of PVT neurons that project to the NAcSh mimics morphine's effects on the acquisition of context associations (Fig. 3). However, it is known that ventromedial NAcSh-projecting PVT neurons (our targeted region) bifurcate sending collateral projections to brain regions involved in the acquisition of context associations like the central amygdala (Dong et al., 2017; Li et al., 2021). Therefore, when inhibiting PVT neurons that project to the NAcSh, we were also potentially inhibiting collateral projections to other brain regions, thus contributing to a robust increase in place preference. Future studies will need to investigate these projection-specific effects.

Interestingly, although we did see a significant increase in the CPP score during somatic inhibition of PVT neurons that project to the NAcSh (Fig. 3), this outcome was not observed when we directly inhibited PVT presynaptic terminals in the NAcSh (Fig. 5). These results suggest that acquisition of CPP, which entails, in part, learning and memory and reward, is not solely dependent upon PVT-to-NAcSh signaling but may require the inclusion of other PVT signaling pathways in addition to or in the absence of PVT-to-NAcSh signaling. Prior studies have demonstrated that chemogenetic inhibition of the midline PVT-to-NAcSh pathway in conjunction with systemic morphine exposure does not prevent the acquisition of morphine-induced CPP (Keyes et al., 2020). However, the interpretation of these findings remains challenging, as it is unclear whether the observed lack of effect is due to an occlusion effect (chemogenetic inhibition is ineffective due to concurrent inhibition by morphine) or if PVT-to-NAcSh neurotransmission is genuinely not required for the acquisition of morphine-induced CPP. Our findings suggest that midline PVT neurotransmission in the NAcSh is not required for the acquisition of CPP. Despite this, there does appear to be a key role of PVT-to-NAcSh signaling in motivational states. It was shown that chemogenetic inhibition of midline PVT-to-NAcSh neurotransmission following the acquisition of morphine CPP (i.e., conditioning sessions) blocked the expression of morphine-induced CPP (Keyes et al., 2020). Additionally, it was shown that activation of posterior PVT-to-NAcSh was sufficient to drive heroin reinstatement after abstinence (Giannotti et al., 2021). Interestingly, midline PVT-to-NAc stimulation promotes wakefulness (Ren et al., 2018), and blocking dopamine-induced neuromodulation in the NAc abolishes the locomotor effects of opioids (Stinus et al., 1980; Kalivas et al., 1983). Based on our findings and those of others, it is plausible to speculate that PVT-to-NAcSh neurotransmission along with neuromodulation by dopamine promotes arousal, which is required for reward-seeking behaviors.

There is another possible explanation for why inhibiting PVT terminals in the NAcSh was not sufficient to evoke CPP (Fig. 5). We observed a significant difference in the variance of the CPP score between mice with inhibition of PVT neurons that project to the NAcSh (Fig. 3) versus mice with inhibition of PVT presynaptic terminals in the NAcSh (Fig. 5). The significant increase in the CPP score's variance in mice with inhibition of PVT presynaptic terminals in the NAcSh suggests that there is a heterogeneous neuronal population that contributes to various facets of the behavioral output. It is known that the PVT sends projections to the NAcSh that target different neuronal populations, including D1-MSNs, D2-MSNs, and interneurons, including parvalbumin interneurons (Zhu et al., 2016; Vollmer et al., 2022). D1-MSNs and D2-MSNs both innervate the ventral pallidum, however, D1-MSNs innervate the lateral hypothalamus, ventral tegmental area, and substantia nigra (Heimer et al., 1991; Usuda et al., 1998; Zhou et al., 2003; Tripathi et al., 2010; O'Connor et al., 2015; Yang et al., 2018). Likewise, interneurons within the NAcSh are well-positioned to modulate both D1-MSN and D2-MSN function and regulate behavioral outcomes (Witten et al., 2010; Brown et al., 2012; Qi et al., 2016; Yu et al., 2017). These connectivity discrepancies potentially explain why inhibition of PVT presynaptic terminals in the NAcSh, in the absence of direct cell-type specific manipulations, results in heterogeneous behavioral outcomes when measuring the acquisition of reward-related context associations. In line with this, microinjections of morphine into the NAc have yielded inconsistent findings, as one study reported place preference (van der Kooy et al., 1982), while another indicated no such preference (Olmstead and Franklin, 1997). These contrasting results may be due to opioid-induced inhibition of specific regions in the NAc or neuronal populations.

Based on our findings and those of others, it is tempting to simplify the role of the PVT in reward processing by stating that when activated, the PVT evokes aversive behaviors and when inhibited, the PVT generates behaviors associated with positive reinforcement. However, reward processing encompasses a multitude of complex neural and behavioral functions that are regulated by the PVT, including *arousal/wakefulness* (Gent et al., 2018; Ren et al., 2018; Wang et al., 2021; Eacret et al., 2023), *stress* (Penzo et al., 2015; Öz et al., 2017; Bengoetxea et al., 2020; Dong et al., 2020; Yu et al., 2021; Corbett et al., 2022a,b), *learning and memory* (Hamlin et al., 2009; Li et al., 2011; Browning et al., 2014; Haight et al., 2015; Otis et al., 2017, 2019; Keyes et al., 2020), *prediction* (Munkhzaya et al., 2020), and *reinforcement* (Marchant et al., 2010; Matzeu et al., 2015; Labouèbe et al., 2016; Zhang and van den Pol, 2017; Cheng et al., 2018; Giannotti et al., 2018, 2021; Kuhn et al., 2018; Campus et al., 2019; Lafferty et al., 2020; Matzeu and Martin-Fardon, 2020; Chisholm et al., 2021; Kessler et al., 2021; Vollmer et al., 2022; Brown and Chaudhri, 2023). Therefore, it is more likely that the role of the PVT in reward processing is nuanced and multifaceted, depending upon the specific stage of reward learning, the type of reward (natural or drug-related), the PVT region (e.g., anterior, middle, posterior), the PVT cell type (Gao et al., 2023), and the brain regions that the PVT interacts with. This has been elegantly demonstrated by a recent study showing the role that the PVT plays in mediating the balance between behaviors associated with seeking reward and those associated with avoiding danger (Choi and McNally, 2017). The study found that the chemogenetic suppression of the PVT results in a behavioral bias toward either defensive or rewarding responses, depending on the specific experimental conditions, without consistently favoring one response over the other (Choi and McNally, 2017). These results support the complex role of the PVT in regulating the balance between behaviors associated with seeking rewards and those associated with avoiding danger.

### Limitations

Our findings do not come without limitations. First, although female mice were used throughout the study, we were unable to include sufficient numbers of female mice to perform statistical comparisons between males and females. Second, the discrepancies observed between somatic inhibition of PVT neurons that project the NAcSh (Fig. 3) versus PVT terminal inhibition in the NAcSh (Fig. 5) are potentially explained by technical limitations. For example, off-target CNO DREADD(Gi) activation of PVT terminals in the NAc core may have contributed to our nonsignificant findings (Fig. 5). However, this is unlikely as PVT neurons that project to the NAc core are less numerous than those to the NAcSh (Dong et al., 2017) and our trypan blue procedure showed minimal NAc core staining. Additionally, evidence suggests that the nonspecific effects can occur from specific AAV serotypes (Haery et al., 2019). For example, AAV1 and AAV9 demonstrate anterograde transsynaptic transport at high titers (Zingg et al., 2017). Here, an AAV2-retro–engineered serotype was employed with no evidence of this serotype possessing anterograde transport. Therefore, it is unlikely that any nonspecific transport took place in the data presented in Figure 3. However, if nonspecific transport were to take place, guide cannula–directed CNO injections targeting the PVT would minimize any potential confounds caused by nonspecific transport. Lastly, our investigation focused on midline PVT projections to the NAcSh and did not investigate the effects of the anterior or posterior PVT in the formation and expression of context associations. These investigations are required in order to fully comprehend an understanding of PVT function.

## Conclusions

It is known that the acquisition of drug–context associations relies on the coordinated activity of many different brain regions, including those involved in signaling salient cues (ventral tegmental area and nucleus accumbens), contributing to affective, emotion, and cognitive control (amygdala, insula, prefrontal cortex, and anterior cingulate cortex), signaling sensation (somatosensory cortex), and processing spatial information and memory (hippocampus; McKendrick and Graziane, 2020). These results establish the PVT as a brain region situated within a complex neurocircuit that mediates the acquisition of opioid context associations and provide evidence that inhibition of PVT neurons is associated with reward.

Overall, this is one of the first studies to examine the direct effects of PVT inhibition on reward-related behaviors through manipulations related to drugs of abuse (morphine) and through chemogenetic approaches that mimic morphine-induced effects. Future experiments could manipulate specific PVT projection neurons, including those that express MORs, and identify behavioral outcomes following either activation or inhibition of these neuronal subtypes. These continued manipulations of PVT neurons will help guide our understanding of the dynamic activity of the PVT as it integrates signals related to reward, danger, and arousal.

## Synthesis

Reviewing Editor: Michael Michaelides, NIDA-NIH Decisions are customarily a result of the Reviewing Editor and the peer reviewers coming together and discussing their recommendations until a consensus is reached. When revisions are invited, a fact-based synthesis statement explaining their decision and outlining what is needed to prepare a revision will be listed below. The following reviewer(s) agreed to reveal their identity: Shelly Flagel. Note: If this manuscript was transferred from JNeurosci and a decision was made to accept the manuscript without peer review, a brief statement to this effect will instead be what is listed below.

Reviewer 1

This manuscript has improved from its previous version, but some concerns remain, as indicated below.

With the incorporation of the Keyes et al., 2020 manuscript in the Introduction, it is unclear how the reported findings add to that literature. It is stated that "...it remains unknown whether direct inhibition of the PVT-to-NAc pathway in the absence of other stimuli is sufficient to promote reward", but it seems that this is also what Keyes and co-authors addressed.

Further discussion/justification for targeting the mid-PVT and its projections to the NAc is warranted. How do the neuroanatomical coordinates targeted here compare to those in the Keyes et al., 2020, manuscript and how might the similarities or differences affect the interpretation of the findings. Why weren't the anterior or posterior subdivisions of the PVT targeted?

It remains unclear why only the 50 ng dose of morphine was given in combination with CTAP.

For the statistics supporting Figure 1 in the main text, it should be noted that the experimental design prevented the direct comparison of Mor vs. Mor + CTAP to aCSF and that the percent change comparison is the best manner to make this comparison. Further, to support the statements made regarding the increases or decreases (or lack thereof) with respect to percent change, a one-sample t-test (with respect to zero) should be conducted for each experimental group.

The number of different animals included in the final neuron count in Figure 1 should be specified.

Male and female mice/samples are not identified in Figures 1, 2 or 4.

For the description of Figure 1h in the main text, it is unclear if the reported statistics are in reference to an overall effect of current or an interaction between current and group. Further, it is difficult to decipher which color circles correspond to which groups in Figure 1h.

Cohen's d should be reported for all statistics in the main text, where appropriate (i.e., all pairwise comparisons).

Did the effect of 50 ng of morphine differ from that of 500 ng or 5 ug, as shown in Figure 2b?

The reported statistics should correspond with the data shown in Figure 2d. It appears that statistics are only reported for the conditioned chamber, yet both chambers are shown in the Figure. Further, it is difficult to distinguish between the colors used for the 50 vs 500 ng groups.

Additional statistics need to be reported for Figure 3C, as it isn't clear what the two-way ANOVA in the main text is referring to. The effects of virus (mCherry vs DREADD) and treatment (sal vs CNO), and interactions should be reported with appropriate post-hoc comparisons and effect sizes.

Given the attention to the data shown in Supplemental Figure 4, this should be moved to the main text.

Additional statistics need to be reported for Figure 5C, as it isn't clear what the two-way ANOVA in the main text is referring to. The effects of virus (mCherry vs DREADD) and treatment (sal vs CNO), and interactions should be reported with appropriate post-hoc comparisons and effect sizes.

Given that there was no evidence for morphine-induced sensitization in any of the experimental groups in the current study, the authors cannot necessarily conclude that the PVT is not involved in sensitization. Further, the lack of a locomotor or sensitization effect with the current paradigm should be discussed with respect to the reported findings.

The statement included in lines 435-438 should be moved to the Results and the findings then referenced in the Discussion.

## Author Response

We again thank the reviewers for their helpful comments that we feel have strengthened our revised manuscript. Below, we respond to each concern raised by the reviewers in italics and have made the corresponding corrections in red font throughout the manuscript.

Comment 1: Extended Data should be labeled as Figure 1-1, Figure 1-2, Table 1-1, etc., so they indicate which figure they are supporting (i.e. Extended Data table supporting Figure 5 labeled as Figure 5-1). This change is not necessary at this stage, but will be if your paper receives a revise decision.

Response: Extended data are now labeled correctly and referenced in the main text.

Comment 2: With the incorporation of the Keyes et al., 2020 manuscript in the Introduction, it is unclear how the reported findings add to that literature. It is stated that "...it remains unknown whether direct inhibition of the PVT-to-NAc pathway in the absence of other stimuli is sufficient to promote reward", but it seems that this is also what Keyes and co-authors addressed.

Response: Our investigation differs from Keyes et al., in two distinct and particularly important ways. First, we directly injected morphine within the PVT to investigate the effects of localized central effects of morphine on the formation of context associations. Keyes et al., employed systemic intraperitoneal injections of morphine, which targets peripheral and central physiological systems. Second, we conditioned mice by employing direct PVT inhibition using DREADDs. This was performed in the absence of morphine. Keyes et al., conditioned mice with systemic morphine injections, while simultaneously inhibiting PVT using DREADDs. Therefore, Keyes et al. manipulated PVT activity in the presence of systemic morphine injections, such that all conditioning sessions were dependent upon morphine administration.

Our findings provide a very distinct and important contribution to the field whereby we investigated whether inhibiting PVT projections to the NAcSh is involved in the formation of context associations. This was performed in the absence of other drugs (e.g., morphine). We have made this distinction in the Discussion section and highlight how our findings add a critical piece of information to our understanding of PVT function (lines 454-458).

Comment 3: Further discussion/justification for targeting the mid-PVT and its projections to the NAc is warranted. How do the neuroanatomical coordinates targeted here compare to those in the Keyes et al., 2020, manuscript and how might the similarities or differences affect the interpretation of the findings. Why weren't the anterior or posterior subdivisions of the PVT targeted?

Response: Keyes et al., targeted midline PVT (from Bregma: AP: -1.4 mm) similar to our investigation. In the Discussion, we have included these important distinctions in PVT coordinates in the studies that were discussed.

The anterior and posterior subdivisions of the PVT were not targeted because we do not have the resources available to perform such an extensive study at this time. This has been added to the limitation section.

Comment 4: It remains unclear why only the 50 ng dose of morphine was given in combination with CTAP.

Response: A significant increase in the CPP score was observed ONLY following 50 ng of morphine injected directly in the PVT; neither the 5 nor 500ng dose elicited a preference (Fig 2B). To confirm that this significant increase was mediated by µ-opioid receptors, we ran an extra set of experiments with 50 ng of morphine in the presence of CTAP. The experiment was designed to determine if CTAP blocked morphine preference, therefore we did not administer CTAP to mice that had no preference following 5 or 500ng morphine.

Comment 5: For the statistics supporting Figure 1 in the main text, it should be noted that the experimental design prevented the direct comparison of Mor vs. Mor + CTAP to aCSF and that the percent change comparison is the best manner to make this comparison. Further, to support the statements made regarding the increases or decreases (or lack thereof) with respect to percent change, a one-sample t-test (with respect to zero) should be conducted for each experimental group.

Response: We have included a statement in the Results that we feel best represents the data shown and provides appropriate data analysis to make an accurate interpretation (lines 249-252).

Comment 6: The number of different animals included in the final neuron count in Figure 1 should be specified.

Response: This is now included in the figure legend.

Comment 7: Male and female mice/samples are not identified in Figures 1, 2 or 4.

Response: This is now included in the figure legends.

Comment 8: For the description of Figure 1h in the main text, it is unclear if the reported statistics are in reference to an overall effect of current or an interaction between current and group. Further, it is difficult to decipher which color circles correspond to which groups in Figure 1h.

Response: This is an interaction term (line 233). Only interaction terms are reported for analyses using a 2-way ANOVA unless otherwise stated. Color circles have been made clearer in Figure 1H.

Comment 9: Cohen's d should be reported for all statistics in the main text, where appropriate (i.e., all pairwise comparisons).

Did the effect of 50 ng of morphine differ from that of 500 ng or 5 ug, as shown in Figure 2b?

Response: Cohen's d values are now reported in the main text.

There were no significant differences between 50 ng morphine versus 500 ng or 5 ug using a Bonferroni posttest. A significant difference was only observed between 50 ng morphine versus saline groups.

Comment 10: The reported statistics should correspond with the data shown in Figure 2d. It appears that statistics are only reported for the conditioned chamber, yet both chambers are shown in the Figure. Further, it is difficult to distinguish between the colors used for the 50 vs 500 ng groups.

Response: Figure 2A shows the CPP apparatus to illustrate the distinct chambers that are used in the conditioning session. Our protocol uses a biased design, in which morphine or saline is paired with the least preferred side of the chamber. Saline should have no impact and as shown in Fig 2B, animals do not distinguish between either side of the apparatus. In contrast, animals conditioned with morphine at the 50ng dose prefer to spend time on this side compared to their original preferred side; CPP score = time on initial preferred side -time on drug/saline conditioned side). Statistics are reported for the CPP score.

The colors have now been updated.

Comment 11 and 12: Additional statistics need to be reported for Figure 3C, as it isn't clear what the two-way ANOVA in the main text is referring to. The effects of virus (mCherry vs DREADD) and treatment (sal vs CNO), and interactions should be reported with appropriate post-hoc comparisons and effect sizes. Comment 12: Additional statistics need to be reported for Figure 5C, as it isn't clear what the two-way ANOVA in the main text is referring to. The effects of virus (mCherry vs DREADD) and treatment (sal vs CNO), and interactions should be reported with appropriate post-hoc comparisons and effect sizes.

Response: These are interaction terms. Based on previous reviewer comments we report interaction terms for Figures 3 and 5 because it informs us whether changes in the CPP score are dependent upon the interaction between the virus and the treatment, which is our research focus in addition to the pairwise comparisons. We opted not to include the main effects of viruses or treatment in the main text to maintain a clear and focused investigation without introducing unnecessary complexity. However, we have included that information in the Extended data (Figures 3-4 and 5-4).

Comment 13: Given the attention to the data shown in Supplemental Figure 4, this should be moved to the main text.

Response: This has been moved to the main text.

Comment 14: Given that there was no evidence for morphine-induced sensitization in any of the experimental groups in the current study, the authors cannot necessarily conclude that the PVT is not involved in sensitization. Further, the lack of a locomotor or sensitization effect with the current paradigm should be discussed with respect to the reported findings.

Response: We have now addressed this in the Discussion (lines 430-439). Briefly, we and others observe locomotor sensitization following systemic injections of morphine in C57BL/6 mice. Thus, we are confident in our ability to measure locomotor sensitization. We do address the possibility that context may matter, however, the Keyes et al 2020 study also observed no effect on locomotor sensitization when they inhibited PVT to amygdala projections. We did not observe any changes in locomotor activity following local injections of morphine in the PVT. Combine this with other findings, now Discussed (lines 430-439), and the results suggest that the PVT and its projections on their own are not sufficient to generate morphine-induced locomotor sensitization.

Comment 15: The statement included in lines 454-458 should be moved to the Results and the findings then referenced in the Discussion.

Response: This has been moved to the Results section (lines 380-385).
