# Erratum: McDevitt et al., “The Paraventricular Thalamic Nucleus and Its Projections in Regulating Reward and Context Associations”

**DOI:** 10.1523/ENEURO.0570-24.2024

**Published:** 2025-01-17

In the article, “The Paraventricular Thalamic Nucleus and Its Projections in Regulating Reward and Context Associations,” by Dillon S. McDevitt, Quinn W. Wade, Greer E. McKendrick, Jacob Nelsen, Mariya Starostina, Nam Tran, Julie A. Blendy, and Nicholas M. Graziane which was published online on February 13, 2024, numerous units of measurement appeared incorrectly due to a production error. Micromolar (µM) was changed to micrometers (µm) throughout the paper. Additionally, the abbreviation for nanoliters was incorrectly formatted as “nl” instead of “nL,” and milliliters was incorrectly abbreviated as “ml” rather than the correct “mL.” These changes do not affect the conclusions of the paper, and the online version has been corrected.

