# Supplementary figures and images for: The Paraventricular Thalamic Nucleus and Its Projections in Regulating Reward and Context Associations
Source: eNeuro. 2024 Feb 9;11(2):ENEURO.0524-23.2024. doi: 10.1523/ENEURO.0524-23.2024 (PMC10883411; doi:10.1523/ENEURO.0524-23.2024)

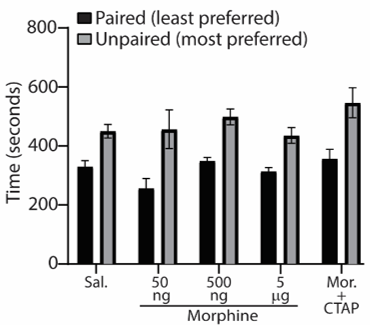

Supplement: Figure 2-1 — Summary graph of the CPP data in Figure 2 showing the average time spent on the drug-paired chamber (least preferred) and control-paired chamber (most preferred) during two habituation sessions (saline: n = 20; morphine (50 ng): n = 15; morphine (500 ng): n = 7; morphine (5 μg): n = 8; morphine (50 ng) + CTAP: n = 8). Download Figure 2-1, TIF file. [file eneuro-11-ENEURO.0524-23.2024-s002.tif]

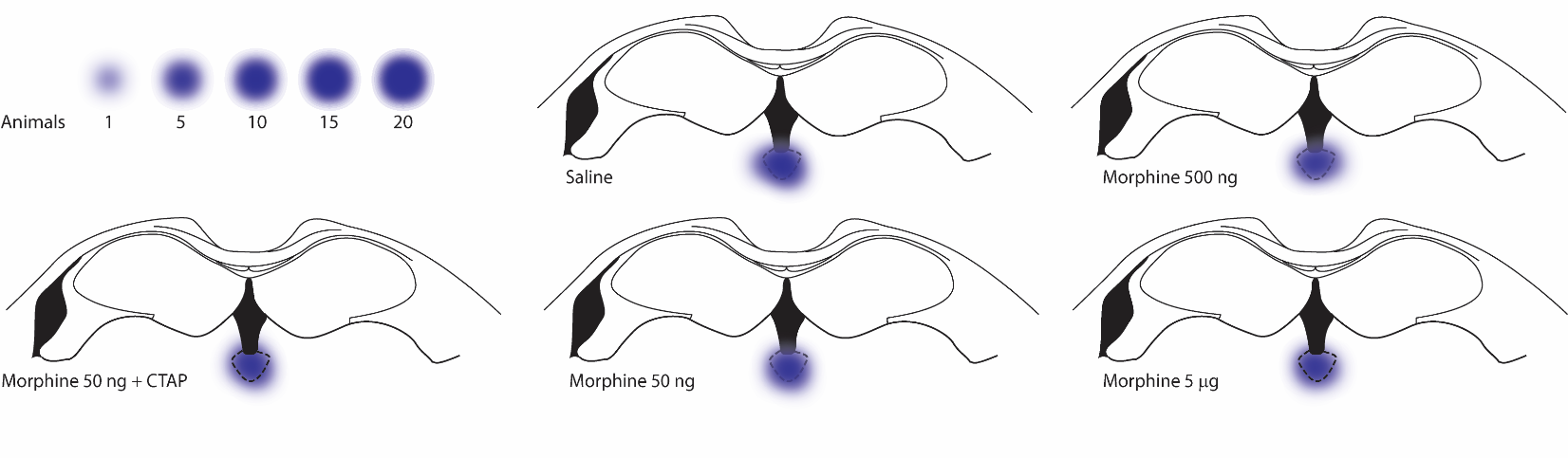

Supplement: Figure 2-2 — Summary of PVT cannula placements for mice undergoing CPP with direct morphine injections into the PVT. The figure represents Evans Blue staining of individual animals. Intensity of the stain is based on the number of animals with an N of 1 corresponding to low intensity and N of 20 the maximum intensity. Download Figure 2-2, TIF file. [file eneuro-11-ENEURO.0524-23.2024-s003.tif]

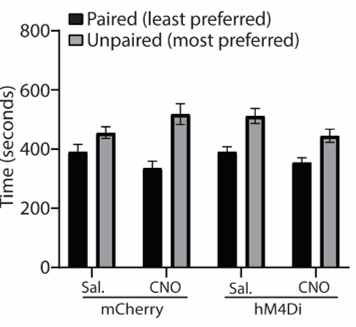

Supplement: Figure 3-1 — Extended data summary of Figure 3 CPP data showing the average time spent on the drug-paired chamber (least preferred) and control-paired chamber (most preferred) during two habituation sessions (mCherry-sal: n = 8; mCherry-CNO: n = 10; hM4Di-sal: n = 9; hM4Di-CNO: n = 8). Download Figure 3-1, TIF file. [file eneuro-11-ENEURO.0524-23.2024-s004.tif]

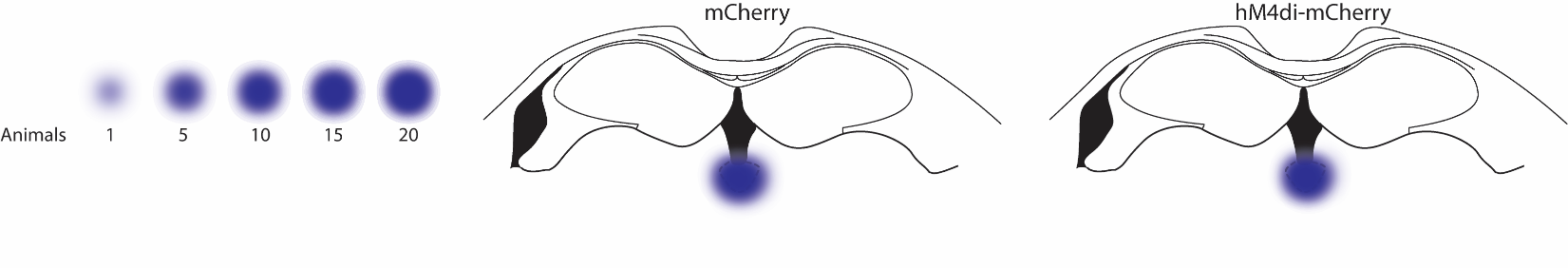

Supplement: Figure 3-2 — Extended data summary of PVT cannula placements for mice undergoing CPP with direct CNO injections into the PVT. The figure represents Evans Blue staining of individual animals. Intensity of the stain is based on the number of animals with an N of 1 corresponding to low intensity and N of 20 the maximum intensity. Download Figure 3-2, TIF file. [file eneuro-11-ENEURO.0524-23.2024-s005.tif]

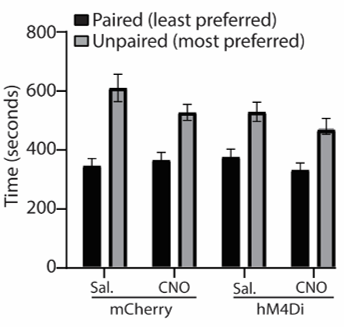

Supplement: Figure 5-1 — Extended data summary graph of Figure 5 CPP data showing the average time spent on the drug-paired chamber (least preferred) and control-paired chamber (most preferred) during two habituation sessions (mCherry-sal: n = 12; mCherry-CNO: n = 12; hM4Di-sal: n = 10; hM4Di-CNO: n = 8). Download Figure 5-1, TIF file. [file eneuro-11-ENEURO.0524-23.2024-s006.tif]

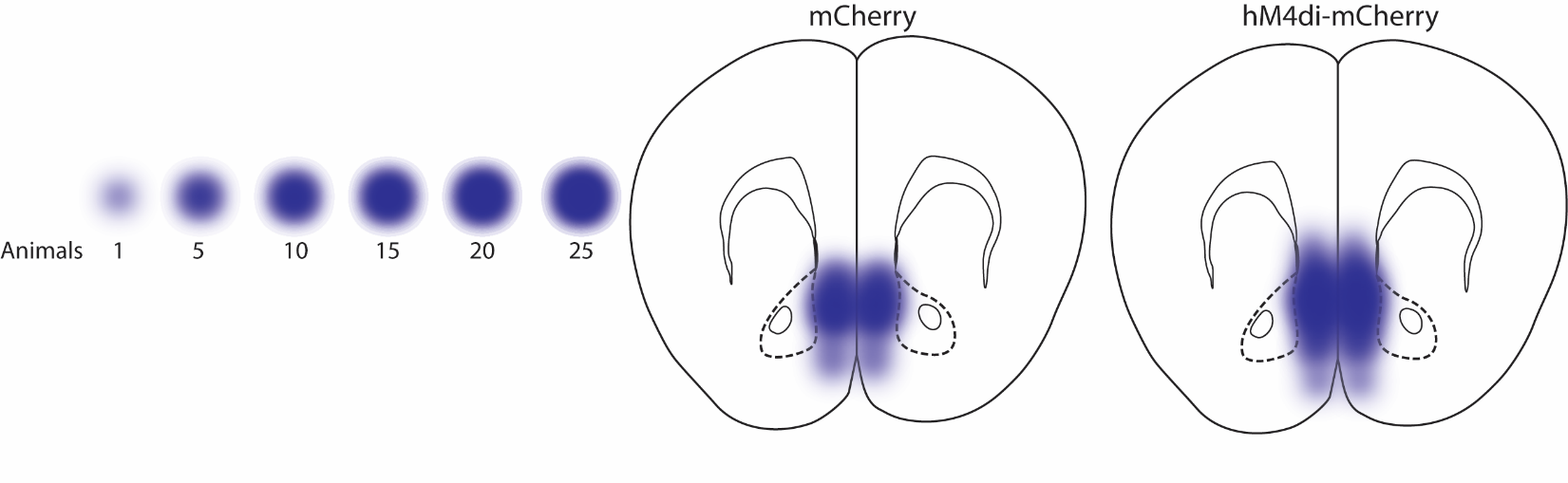

Supplement: Figure 5-2 — Extended data summary of NAcSh cannula placements for mice undergoing CPP with direct CNO injections into the NAcSh. The figure represents Evans Blue staining of individual animals. Intensity of the stain is based on the number of animals with an N of 1 corresponding to low intensity and N of 25 the maximum intensity. Download Figure 5-2, TIF file. [file eneuro-11-ENEURO.0524-23.2024-s007.tif]
