# Supplementary material for: The Paraventricular Thalamic Nucleus and Its Projections in Regulating Reward and Context Associations
Source: eNeuro. 2024 Feb 9;11(2):ENEURO.0524-23.2024. doi: 10.1523/ENEURO.0524-23.2024 (PMC10883411; doi:10.1523/ENEURO.0524-23.2024)
Supplement: Table 2-3 — Extended data table providing effect size comparisons for CPP data supporting Figure 2. Download Table 2-3, DOC file. [file eneuro-11-ENEURO.0524-23.2024-s008.doc]

| **Figure 2-3** | | |
| --- | --- | --- |
| **Comparison** | ***p*-value** | **Absolute Cohen’s *d*** (mean_1_-mean_2_/SD_pooled_) |
| saline vs. morphine(50 ng) | 0.0485* | 1.92 |
| saline vs. morphine(500 ng) | 0.9280 | 0.829 |
| saline vs. morphine(5 μg) | 0.2661 | 0.857 |
| morphine(50 ng) vs. morphine(500 ng) | >0.9999 | 0.583 |
| morphine(50 ng) vs. morphine(5 μg) | >0.9999 | 0.451 |
| morphine(500 ng) vs. morphine(5 μg) | >0.9999 | 0.065 |

**p* < 0.05

Cohen’s *d:* Small effect: 0.2 ≤ *d* ≤ 0.49

Medium effect: 0.5 ≤ *d* ≤ 0.79

Large effect: *d* ≥ 0.80
