# Supplementary material for: The Paraventricular Thalamic Nucleus and Its Projections in Regulating Reward and Context Associations
Source: eNeuro. 2024 Feb 9;11(2):ENEURO.0524-23.2024. doi: 10.1523/ENEURO.0524-23.2024 (PMC10883411; doi:10.1523/ENEURO.0524-23.2024)
Supplement: Table 5-3 — Extended data table providing effect size comparisons for CPP data supporting Figure 5. Download Table 5-3, DOC file. [file eneuro-11-ENEURO.0524-23.2024-s011.doc]

| **Figure 5-3** | | |
| --- | --- | --- |
| **Comparison** | ***p*-value** | **Absolute Cohen’s *d*** (mean_1_-mean_2_/SD_pooled_) |
| mCherry(sal) vs. mCherry(CNO) | >0.9999 | 0.050 |
| mCherry(sal) vs. hM4Di(sal) | >0.9999 | 0.204 |
| mCherry(sal) vs. hM4Di(CNO) | 0.5417 | 0.983 |
| mCherry(CNO) vs. hM4Di(sal) | >0.9999 | 0.291 |
| mCherry(CNO) vs. hM4Di(CNO) | 0.6626 | 1.17 |
| hM4Di(sal) vs. hM4Di(CNO) | 0.2312 | 1.225 |

Cohen’s *d:* Small effect: 0.2 ≤ *d* ≤ 0.49

Medium effect: 0.5 ≤ *d* ≤ 0.79

Large effect: *d* ≥ 0.80
