# Supplementary material for: The Paraventricular Thalamic Nucleus and Its Projections in Regulating Reward and Context Associations
Source: eNeuro. 2024 Feb 9;11(2):ENEURO.0524-23.2024. doi: 10.1523/ENEURO.0524-23.2024 (PMC10883411; doi:10.1523/ENEURO.0524-23.2024)
Supplement: Table 5-4 — Extended data table supporting Figure 5 showing main effects of virus (mCherry and hM4Di) and treatment (sal and CNO). Download Table 5-4, DOC file. [file eneuro-11-ENEURO.0524-23.2024-s012.doc]

| **Figure 5-4** | | |
| --- | --- | --- |
| **Main effect** | ***F* value** | ***p*-value** |
| Virus (mCherry vs. hM4Di) | *F*_(1,38)_ = 0.6972 | 0.4089 |
| Treatment (saline vs. CNO) | *F*_(1,38)_ = 2.891 | 0.0972 |
